# Enhancing WHO prescription writing guideline adherence through an educational intervention: a quality improvement study of Azad Jammu and Kashmir

**DOI:** 10.1097/JS9.0000000000001828

**Published:** 2024-06-13

**Authors:** Mushood Ahmed, Areeba Ahsan, Muhammad Selal Zia, Qura Tul Ain, Salwa Khurshid, Jawad Basit, Tagwa Kalool Fadlalla Ahmad

**Affiliations:** a Department of Medicine, Rawalpindi Medical University, Rawalpindi; b Department of Medicine, Foundation University School of Health Sciences; c Department of Pharmacology, Shifa Tameer e Millat University, Islamabad; d Department of Medicine, Gomal Medical College, Dera Ismail Khan, Pakistan; e Cardiovascular Analytics Group, Canterbury, UK; f Ahfad University for Women, Omdurman, Sudan

**Keywords:** adherence, audit, education, prescriptions, WHO

## Abstract

**Background:**

Prescription writing is an important component of healthcare delivery and can directly influence patient safety and treatment outcomes. Prescription errors are common in developing countries because of the lack of national guidelines. This two-cycle clinical audit assessed the impact of educational interventions on improving prescription writing practices.

**Methods:**

A cross-sectional prospective clinical audit was conducted in the Out Patients Department (OPD) of the District Head Quarters (DHQ) Hospital in Bhimber, Azad Jammu, and Kashmir. A total of 100 randomly selected prescriptions were reviewed for each cycle from July to August 2023. The authors recorded compliance with WHO guidelines for prescription writing before and after the educational intervention. Microsoft Excel and SPSS version 25.0 were used for statistical analysis. Categorical variables were analyzed using frequencies and percentages.

**Results:**

An improvement in compliance was observed during the second audit cycle, after the educational intervention. The greatest improvement was observed in documenting the allergic status of patients (62%) and the direction of drug administration (40%). The authors also observed improvements in the treatment duration (>10%), patient weight, physician registration number, diagnosis, and follow-up advice. The legibility of prescriptions also improved during the second audit cycle.

**Conclusion:**

This study shows that integrating an educational intervention into a clinical audit can improve prescription writing practices and ultimately result in a better quality of care for patients.

## Introduction

Highlights

- This study represents the first-ever clinical audit conducted in the history of Azad Jammu and Kashmir (AJK), a region with a population of over 4 million.
- The region of AJK has no established guidelines for prescription writing. Considering this literature gap, a pilot clinical audit was conducted to improve prescription writing practices. An educational intervention was performed after the completion of the first audit cycle. The intervention resulted in considerably improved prescription quality during the second audit cycle.
- The proactive implementation of an educational intervention between the first and second audit cycles demonstrates a commitment to continuous improvement.

A prescription is “a written order, which includes detailed instructions of what medicine should be given to whom, in what formulation and dose, by what route, when, how frequently, and for how long”^1^. A prescription serves as a direction by a healthcare professional to provide a specific treatment plan, usually a medication for the patient^2^. It is regarded as a medicolegal document that should adhere to standards and be written accurately and legibly^3^. In developing countries, handwritten prescriptions are the mainstay of communication between healthcare providers and patients^4^.

Physicians should be aware of the elements of prescription in Document^3^. Well-written prescriptions play an important role in reducing errors in the dispensing of medications. Inaccuracies in prescription writing can compromise quality of life and patient safety^5^. These errors are often the result of errors in prescription writing by healthcare professionals^6,7^. These errors contribute to more than 70% of medication errors, ultimately leading to adverse effects^8^. Illegible handwriting is another problem that can lead to difficulty understanding prescriptions^9^.

WHO has set a standard for prescription writing that defines the essential elements of a well-written prescription^10^. These guidelines are used across the world to determine the quality of prescriptions written by healthcare providers^4,11,12^. There is a lack of local guidelines regarding prescription writing in Kashmir, which has resulted in medication errors^13^. Moreover, no study has been conducted in Azad Jammu and Kashmir (AJK) to determine the adherence of physicians’ prescriptions to international standards and improve the quality of prescription writing. Considering this literature gap, we decided to conduct a clinical audit to prospectively assess the compliance of prescriptions to WHO prescription writing guidelines and improve the quality of prescriptions by integrating educational interventions in the audit.

## Methods

A clinical audit was conducted in the Out Patients Internal Medicine Department of District Head Quarters (DHQ) Hospital, Bhimber, AJK. DHQ Bhimber is responsible for providing healthcare services to the whole district’s population of over 0.4 million. Ethical approval for the study was obtained from the medical superintendent of the DHQ Hospital. A clinical audit was conducted following the guidelines established by the Revised Standards for Quality Improvement Reporting Excellence (SQUIRE 2.0) guidelines^14^. The study population comprised patients visiting the OPD of DHQ hospital Bhimber, AJK. The study population comprised patients visiting the OPD of DHQ hospital Bhimber, AJK. The sample size was estimated to be 100 for each audit cycle using a 95% CI, 5% margin of error, and 7% prevalence of incomplete prescriptions based on a pilot study. One hundred prescriptions were reviewed in each audit cycle using simple random sampling. The SQUIRE 2.0 checklist is provided in the Supplementary Appendix S1, Supplemental Digital Content 1, http://links.lww.com/JS9/C742.

The senior researcher (MA) briefed the data collectors about the use of the WHO Guide to Good Prescribing^10^. The same tool has been used in various studies to assess the quality of prescriptions quality and compliance with WHO standards^4,8,15,16^. The essential elements of a well-written prescription, according to the WHO, are as follows:

- Patient identifiers: Name, age, sex, weight
- Prescribing physician identifiers: Name of physician and department, registration number
- Treatment regimen: dosage formulation, strength, direction of administration, duration of treatment, allergic status, and follow-up advice
- Diagnosis
- Legibility of the prescription

The first audit cycle was conducted from 10 July to 25 July 2023. The prescriptions were manually screened by three researchers to evaluate adherence to WHO guidelines. If two of the researchers were unable to read the prescription, it was regarded as ineligible. If one researcher was able to read the prescription, it was regarded as difficult to legible, and if both researchers were able to read the prescription, it was regarded as easily legible. To eliminate the risk of bias, the prescribing physicians were not informed about the audit. All prescriptions were assessed according to WHO standards for a good-written prescription.

After the completion of the first audit cycle, we performed an education intervention from 1 August to 15 August 2023, through panel meetings. Physicians were informed of the importance of following the WHO guide for good prescription. Physicians were advised by the medical superintendent (head of the hospital) to follow the guidelines. A well-written prescription that adhered to the WHO guidelines was distributed in the form of circulars among physicians to enhance their understanding.

The second audit cycle was conducted three weeks after the educational intervention, from 5 to 31^st^ September 2023. We followed the same data collection procedure during the second audit cycle. IBM SPSS Statistics for (version 25.0, IBM Corp) was used for statistical analysis. Categorical variables were analyzed using frequency and percentages. The bar graph was generated using Microsoft Excel.

## Results

A total of 100 prescriptions were analyzed during the first audit cycle. None of the prescriptions mentioned the weight of the patient or the registration number of the prescribing physician. The duration of treatment and allergic status of the patient were mentioned in less than 25% of prescriptions. The direction of the drug administration was not mentioned in any prescription. Approximately 80% of the prescriptions were easily legible, the rest were legible with difficulty, and none of the prescriptions were illegible. Table 1 presents the details of first audit cycle.

**Table 1:** Results of first audit cycle showing compliance (%) for different prescription components.

	Percentage of compliance (%)	
Prescription elements	Yes	No
Date mentioned for the prescription	100	0
Patient identifiers		
Name	100	0
Age	100	0
Sex	100	0
Weight	0	100
Prescribing physician identifiers		
Name	100	0
Name of department	100	0
Registration number	0	100
Treatment regimen and diagnosis		
Dosage formulation	100	0
Drug dosage	99	1
Direction of administration	0	100
Duration of treatment	24	76
Allergic status	23	77
Diagnosis	99	1
Follow-up advice	94	6
Legibility		
Legible with ease	79	21
Legible with difficulty	21	79
Illegible	-	-

After the completion of first audit cycle, an educational intervention was performed to improve compliance with WHO guidelines. We then performed second audit cycle in which 100 prescriptions were analyzed. Table 2 presents the details of second audit cycle.

**Table 2:** Results of second audit cycle showing compliance (%) for different prescription components.

	Frequency/percentage of compliance (%)	
Prescription elements	Yes	No
Date mentioned for the prescription	100	0
Patient identifiers		
Name	100	0
Age	100	0
Sex	100	0
Weight	4	96
Prescribing physician identifiers		
Name	100	0
Name of department	100	0
Registration number	1	99
Treatment regimen and diagnosis		
Dosage formulation	100	0
Drug dosage	99	1
Direction of administration	43	57
Duration of treatment	40	60
Allergic status	85	15
Diagnosis	100	0
Follow-up advice	98	2
Legibility		
Legible with ease	87	13
Legible with difficulty	13	87
Illegible	-	-

### Impact of educational intervention

The date of issuance of the prescription, name, age, and sex of the patient were 100% compliant with the WHO guidelines. Moreover, all prescriptions in both audit cycles mentioned the name of the prescriber, department, and dosage formulation.

**Figure 1:**
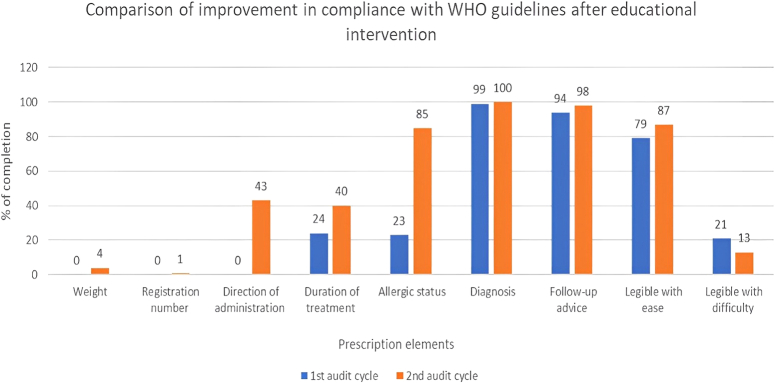
Comparison of improvement in compliance with WHO guidelines after educational intervention.

We observed a marked improvement in the direction of drug administration in second audit cycle compared to the first audit cycle (40% vs. 0%). The allergic status of the patients was mentioned in 85% of the prescriptions during 2nd audit cycle compared to 23% in the first cycle. This indicates an improvement of more than 60% across the two cycles. The treatment duration improved by greater than 10% in the second cycle. We observed a less than 10% improvement in patient weight (0% vs. 4%), registration number of the physician (0% vs. 1%), diagnosis (99% vs. 100%), follow-up advice (94% vs. 98%), and easily legible prescriptions (79% vs. 87%). The % of difficult legible prescriptions also decreased from 21% in first cycle to 13% in the second cycle. The details are shown in Fig. 1.

## Discussion

The initial audit revealed several compliance issues with the WHO prescription writing guidelines at the DHQ and Bhimber AJK. While the issuance date and patient identifiers had full compliance, critical elements such as the patient’s weight and the prescribing physician’s registration number were consistently omitted. Treatment and diagnostic details were lacking, with only a quarter of prescriptions mentioning treatment duration and no mention of the direction of administration. Only 23% of the prescriptions documented the patient’s allergic status, indicating a need for improvement. After the educational intervention, the second audit demonstrated several improvements. Patient identifiers remained in full compliance, and there were improvements in recording patient weights and prescribing physician details. Treatment and diagnostic parameters showed substantial progress, with improvements in the direction of administration and treatment duration. Notably, the allergic status of the patients was better documented, reflecting a stronger commitment to comprehensive and individualized healthcare practices. There was also an overall improvement in prescription legibility, with a higher percentage of prescriptions that were easily readable.

Our study found a concern rate of 100% non-compliance with writing the patient’s weight during the 1st audit cycle. This could be due to the absence of weighing machines at DHQ Bhimber, AJK. The lack of this essential equipment poses a considerable obstacle to adherence to best practices in medication prescription, particularly those that require weight-based dosing. Similar findings from another study revealed 63.47% non-compliance with the patient’s weight^17^. Accurate patient weight is crucial for prescribing drugs, particularly in pediatric patients, emphasizing the necessity of weight-based dosing for optimal medication management^18^. In the current study, we found that 100% of prescriptions included the patients’ name, age, and sex, which is consistent with the findings of Kripalani *et al*.^4^ and Dyasanoor^19^ who also reported good compliance. These results align with WHO’s emphasis on accurate and complete patient information, which is crucial for patient safety and effective healthcare.

We analyzed three prescribing physician identifiers: the prescriber’s name, department name, and prescriber’s registration number. In the first audit cycle, the prescriber’s and department names had a commendable 100% compliance, indicating strong adherence to the guidelines. However, the prescriber registration number was absent. This raises concerns about potential challenges in ensuring the unique identification of prescribers, which is a critical aspect of accountability in healthcare settings. The use of medical license numbers for physician identification in pharmacy claims data is increasingly common for research and quality improvement^20^. Another study reported similar findings, with a 99.6% rate of prescription errors regarding registration numbers^21^. We noticed that in the second audit cycle, there was an improvement in specifying the direction of drug administration, increasing from 0 to 43%, and duration of treatment, increasing from 24 to 40%, after the educational intervention. In comparison, a study of 250 prescriptions found that essential prescription components, such as dose, drug direction, and treatment duration, were inadequately written in 90%, 74%, and 80% of prescriptions, respectively, while doctors’ medical registration numbers were completely omitted^22^. Singh *et al*.^16^ reported that dosing mistakes and neglecting to specify the treatment duration were prevalent, occurring in 26.7% and 27.5% of the cases, respectively.

Our educational interventions led to better documentation of the allergic status of the patients. The inclusion of allergic status in patient records enhances safety and healthcare quality. This commitment to comprehensive healthcare practices reduces the risk of adverse reactions, emphasizing the importance of incorporating known allergy status in medication administration for optimal patient outcomes^23^. Unfortunately, several studies have found that documentation of allergic status in secondary care is suboptimal^24^. Our findings show that prescription legibility markedly improved in the second audit cycle, reflecting a clear enhancement in overall prescription clarity. Improved prescription legibility enhances patient safety, reduces misinterpretation risks, and fosters efficient communication among healthcare providers^4,5,16,19,25^.

Several factors contributed to the low compliance with prescription practices during the first audit cycle. First, physicians’ lack of awareness of the WHO guidelines and the absence of national/local standards created uncertainty. The absence of weighing machines was another contributing factor. Accurate weight measurements, particularly important in pediatric patients, were compromised, affecting the precision of the prescribed dosages. Furthermore, some physicians reported perceived time constraints when adhering to international guidelines for prescription writing. These findings emphasize that faults and errors in the prescription process can often be avoided. Effective intervention strategies should prioritize education and establish a secure and collaborative work environment. This approach aims to reduce potential harm to patients^8^.

This study represents the first clinical audit conducted in the history of Azad Kashmir, a region with a population of over 4 million. The importance of this study extends beyond its historical context and lies in its tangible and transformative results. The proactive implementation of an educational intervention between the first and second audit cycles demonstrates a commitment to continuous improvement.

However, the clinical audit was conducted at the DHQ, Bhimber AJK, which may limit the generalizability of the findings to other healthcare facilities with different practices and resources. The absence of weighing machines at DHQ Bhimber, AJK, during the initial audit cycle may have influenced non-compliance with patient weight documentation. Moreover, there is an urgent need to establish guidelines for prescription writing.

## Conclusion

Our research highlights the significance of offering training to physicians regarding standardized prescription writing so that they can draft prescriptions according to WHO guidelines. Although improvements were observed in certain aspects, such as legibility and documentation of allergic status, further efforts are needed to address persistent challenges, including the inclusion of essential patient information and prescriber identifiers.

## Ethical approval

Ethical approval of the study was obtained from the institutional review board of District Head Quarter Hospital, Bhimber, Azad Kashmir supervised by medical superintendent on 17 June 2023.

## Consent

Consent was taken from patients to review their prescriptions.

## Source of funding

No financial support was received.

## Author contribution

Conceptualization, data curation, and project administration were carried out by M.A., T.K.F.A. and J.B. Supervision was carried out by J.B. Formal analysis of data was carried out by M.A., T.K.F.A. and A.A. Formal analysis, methodology, and software was carried out by M.A., A.A. and S.K. Writing the original draft was carried out by M.A., A.A., Q.T.A. and J.B. Writing, reviewing, and editing were carried out by M.A., A.A. and M.S.Z. Visualization and validation were carried out by M.S.Z., Q.T.A. and S.K.

## Conflicts of interest disclosure

The authors declare no conflict of interest.

## Research registration unique identifying number (UIN)

The research is registered with Azad Jammu and Kashmir Health Department (https://health.ajk.gov.pk/) with UIN of: CAQIP654867.

## Guarantor

Mushood Ahmed.

## Data availability statement

Datasets generated during and/or analyzed during the current study are available upon reasonable request to corresponding author.

## Provenence and peer review

Not invited.

## Supplementary Material

SUPPLEMENTARY MATERIAL
