# Supplementary material for: Enhancing WHO prescription writing guideline adherence through an educational intervention: a quality improvement study of Azad Jammu and Kashmir
Source: Int J Surg. 2024 Jun 13;110(10):6617–21. doi: 10.1097/JS9.0000000000001828 (PMC11487047; doi:10.1097/JS9.0000000000001828)
Supplement: SUPPLEMENTARY MATERIAL [file js9-110-6617-s001.docx]

**Supplementary Appendix**

| **S1: Research and reporting methodology** |  | |  |
| --- | --- | --- | --- |
| Revised **Standards for QUality Improvement Reporting Excellence** (**SQUIRE 2.0**) publication guidelines |  | |  |
|  |  | |  |
|  |  | |  |
|  |  | |  |
| **Text section and item name** | | **Page/line no(s).** | |
|  | | **info is located** | |
| **Title and abstract** | |  | |
| 1. **Title** | |  | |
| Indicate that the manuscript concerns an initiative to improve healthcare (broadly defined to include the quality, safety, effectiveness, patient-centredness, timeliness, cost, efficiency and equity of healthcare). | | Title Page | |
|  | |  | |
| 2. **Abstract** | |  | |
| a. Provide adequate information to aid in searching and indexing. | | 1 | |
| b. Summarise all key information from various sections of the text using the abstract format of the intended publication or a structured summary such as: background, local problem, methods, interventions, results, conclusions. | | 1 | |
|  | |  | |
| **Introduction: Why did you start?** | |  | |
| 3. **Problem description** - Nature and significance of the local problem. | | 3 | |
| 4. **Available knowledge** - Summary of what is currently known about the problem, including relevant previous studies. | | 3 | |
| 5. **Rationale** - Informal or formal frameworks, models, concepts and/or theories used to explain the problem, any reasons or assumptions that were used to develop the intervention(s) and reasons why the intervention(s) was expected to work | | 3-4 | |
| 6. **Specific aims** - Purpose of the project and of this report. | | 3-4 | |
|  | |  | |
| **Methods: What did you do?** | |  | |
| 7. **Context** - Contextual elements considered important at the outset of introducing the intervention(s). | | 4 | |

| 8. **Intervention(s)** |  |
| --- | --- |
| a. Description of the intervention(s) in sufficient detail that others could reproduce it. | 4 |
| b. Specifics of the team involved in the work. | 4 |
| 9. **Study of the intervention(s)** |  |
| a. Approach chosen for assessing the impact of the intervention(s). | 4 |
| b. Approach used to establish whether the observed outcomes were due to the intervention(s). | 4-5 |
| 10. **Measures** |  |
| a. Measures chosen for studying processes and outcomes of the intervention(s), including rationale for choosing them, their operational definitions and their validity and reliability. | 5 |
| b. Description of the approach to the ongoing assessment of contextual elements that contributed to the success, failure, efficiency and cost. | 5 |
| c. Methods employed for assessing completeness and accuracy of data. | 5 |
| 11. **Analysis** |  |
| a. Qualitative and quantitative methods used to draw inferences from the data. | 5 |
| b. Methods for understanding variation within the data, including the effects of time as a variable. | 5 |
| 12. **Ethical considerations** - Ethical aspects of implementing and studying the intervention(s) and how they were addressed, including, but not limited to, formal ethics review and potential conflict(s) of interest. | 4 |
|  |  |
| **Results: What did you find?** |  |
| 13. **Results** |  |
| a. Initial steps of the intervention(s) and their evolution over time (eg, time-line diagram, flow chart or table), including modifications made to the intervention during the project. | 5 |
| b. Details of the process measures and outcomes. | 5-6 |
| c. Contextual elements that interacted with the intervention(s). | 6 |
| d. Observed associations between outcomes, interventions and relevant contextual elements. | 6 |
| e. Unintended consequences such as unexpected benefits, problems, failures or costs associated with the intervention(s). | 6 |
| f. Details about missing data. | 6 |
|  |  |
| **Discussion: What does it mean?** |  |
| 14. **Summary** |  |
| a. Key findings, including relevance to the rationale and specific aims. | 6-7 |
| b. Particular strengths of the project. | 7 |
|  |  |
| 15. **Interpretation** |  |
| a. Nature of the association between the intervention(s) and the outcomes. | 7 |
| b. Comparison of results with findings from other publications. | 7 |
| c. Impact of the project on people and systems. | 7-8 |
| d. Reasons for any differences between observed and anticipated outcomes, including the influence of context. | 8-9 |
| e. Costs and strategic trade-offs, including opportunity costs. | 9 |
|  |  |
| 16. **Limitations** |  |
| a. Limits to the generalisability of the work. | 9 |
| b. Factors that might have limited internal validity such as confounding, bias or imprecision in the design, methods, measurement or analysis. | 9 |
| c. Efforts made to minimise and adjust for limitations. | 9 |
|  |  |
| **Conclusions** |  |
| a. Usefulness of the work. | 10 |
| b. Sustainability. | 10 |
| c. Potential for spread to other contexts. | 10 |
| d. Implications for practice and for further study in the field. | 10 |
| e. Suggested next steps. | 10 |
|  |  |
| **Other information** |  |
| 18. **Funding** - Sources of funding that supported this work. Role, if any, of the funding organisation in the design, implementation, interpretation and reporting. | None |
|  |  |
|  |  |
|  |  |
|  |  |
| *Ogrinc G, et al. BMJ Qual Saf 2015;0:1–7. doi:10.1136/bmjqs-2015-004411* |  |
| *Downloaded from http://qualitysafety.bmj.com/ on January 2, 2017* |  |
